# The safety and efficacy of binimetinib for lung cancer: a systematic review

**DOI:** 10.1186/s12890-024-03178-4

**Published:** 2024-08-01

**Authors:** Mahdi Zahmatyar, Ladan Kharaz, Negin Abiri Jahromi, Ali Jahanian, Pourya Shokri, Seyed Aria Nejadghaderi

**Affiliations:** 1 Student Research Committee, Tabriz University of Medical Sciences, Tabriz, Iran; 2 Faculty of Medicine, Tabriz University of Medical Sciences, Tabriz, Iran; 3 School of Medicine, Shahid Beheshti University of Medical Sciences, Tehran, Iran; 4 Tehran University of Medical Sciences, Tehran, Iran; 5 HIV/STI Surveillance Research Center, WHO Collaborating Center for HIV Surveillance, Institute for Futures Studies in Health, Kerman University of Medical Sciences, Kerman, Iran; 6 Systematic Review and Meta‑analysis Expert Group (SRMEG), Universal Scientific Education and Research Network (USERN), Tehran, Iran

**Keywords:** Binimetinib, Lung neoplasm, Mektovi, MEK inhibitor, Systematic review

## Abstract

**Background:**

Lung cancer, accounting for a significant proportion of global cancer cases and deaths, poses a considerable health burden. Non-small cell lung cancer (NSCLC) patients have a poor prognosis and limited treatment options due to late-stage diagnosis and drug resistance. Dysregulated of the mitogen-activated protein kinase (MAPK) pathway, which is implicated in NSCLC pathogenesis, underscores the potential of MEK inhibitors such as binimetinib. Despite promising results in other cancers, comprehensive studies evaluating the safety and efficacy of binimetinib in lung cancer are lacking. This systematic review aimed to investigate the safety and efficacy of binimetinib for lung cancer treatment.

**Methods:**

We searched PubMed, Scopus, Web of Science, and Google Scholar until September 2023. Clinical trials evaluating the efficacy or safety of binimetinib for lung cancer treatment were included. Studies were excluded if they included individuals with conditions unrelated to lung cancer, investigated other treatments, or had different types of designs. The quality assessment was conducted utilizing the National Institutes of Health tool.

**Results:**

Seven studies with 228 participants overall were included. Four had good quality judgments, and three had fair quality judgments. The majority of patients experienced all-cause adverse events, with diarrhea, fatigue, and nausea being the most commonly reported adverse events of any grade. The objective response rate (ORR) was up to 75%, and the median progression-free survival (PFS) was up to 9.3 months. The disease control rate after 24 weeks varied from 41% to 64%. Overall survival (OS) ranged between 3.0 and 18.8 months. Notably, treatment-related adverse events were observed in more than 50% of patients, including serious adverse events such as colitis, febrile neutropenia, and pulmonary infection. Some adverse events led to dose limitation and drug discontinuation in five studies. Additionally, five studies reported cases of death, mostly due to disease progression. The median duration of treatment ranged from 14.8 weeks to 8.4 months. The most common dosage of binimetinib was 30 mg or 45 mg twice daily, sometimes used in combination with other agents like encorafenib or hydroxychloroquine.

**Conclusions:**

Only a few studies have shown binimetinib to be effective, in terms of improving OS, PFS, and ORR, while most of the studies found nonsignificant efficacy with increased toxicity for binimetinib compared with traditional chemotherapy in patients with lung cancer. Further large-scale randomized controlled trials are recommended.

**Supplementary Information:**

The online version contains supplementary material available at 10.1186/s12890-024-03178-4.

## Introduction

In 2020, lung cancer accounted for 11.6% of all new cancers globally [1]. Approximately 85% of all lung cancer diagnoses, are classified as non-small cell lung cancer (NSCLC), which differs from multiple molecular alterations, particularly lung adenocarcinoma [2–4]. NSCLC is implicated in different pathogeneses and metastatic forms of the disease, with five-year survival rates of less than 5% [2, 5].

Although surgical resection and chemotherapy are still important methods for treating lung cancer, the development of other treatment options for lung cancer is necessary since most cases are diagnosed at advanced stages with local or distant metastases, in addition to the resistance of many advanced NSCLCs to most of clinically applied drugs [6–8]. Therefore, finding new biomarkers, chemical targets, and new therapeutic mechanisms for the treatment of lung cancer is critical [9, 10]. Dysregulation of the mitogen-activated protein kinase (MAPK) pathway involves the mitogen-activated protein kinase enzyme (MEK), extracellular signal-regulated kinase (ERK), RAF, and the RAS signaling cascade; this pathway is involved in approximately one-third of cancers and is involved in the progression and tumorigenesis of a broad array of cancers including NSCLCs [11–15]. Accordingly, various MEK inhibitors in combination with chemotherapy or other targeted agents, are potential therapeutic agents for treating NSCLC [16–26].

Preclinical studies imply that the inhibition of MEK1/2 can be an effective strategy for the treatment of tumors driven by *BRAF* or *KRAS* mutations [27–30]. These mutations result in constitutive activation of the RAS-MAPK signaling pathway, leading to uncontrolled cell growth, proliferation, and survival [31]. Binimetinib (MEK162, ARRY-162, or ARRY-438,162), which is also known as Mektovi, is an orally bioavailable highly selective, and potent non-ATP-competitive allosteric inhibitor and MAPK inhibitor. It was approved in 2018 by the United States Food and Drug Administration for the treatment of patients with *BRAF*-mutant melanoma in the low nanomolar range [25, 32–38]. The single-agent binimetinib has a maximum tolerable dose of 60 mg twice daily (BID) [25]. In addition to its influence on *BRAF*-mutant melanoma, binimetinib has synergistic antitumor clinical activity in tumors like melanoma harboring neuroblastoma-rat sarcoma (NRAS) mutations and Kirsten Rat Sarcoma viral oncogene (KRASm) NSCLCs [25, 35, 38–43].

Moreover, preclinical and clinical evidence supports the efficacy of BRAF and MEK inhibitor combinations in patients with NSCLC among which the combination of MEK162 and buparlisib (BKM120) significantly inhibits tumor growth in epidermal growth factor receptor tyrosine kinase inhibitor (EGFR-TKI) resistant NSCLC cells and overcomes the negative feedback mechanisms in the phosphoinositide 3-kinase (PI3K) pathway [44–46]. Because no comprehensive study has been performed to evaluate the safety and efficacy of binimetinib for application in human subjects, in this systematic review, we aimed to investigate the safety and efficacy of binimetinib in patients with lung cancer.

## Methods

This study was performed according to the Preferred Reporting Items for Systematic Reviews and Meta-Analyses (PRISMA) 2020 guidelines [47].

### Search strategy

We systematically searched PubMed, Scopus, and Web of Science without any search constraints, up to September 15, 2023. A comprehensive combination of the following terms was used in the current study: Binimetinib (e.g. “Binimetinib”, “Mektovi”, “MEK162”, “ARRY162”, “ARRY438162”, “181R97MR71”, “MFCD22124525”, and “CHEMBL3187723”) and terms related to cancer like (“Tumor”, “Cancer”, “Neoplas*”, “Cancer*”, “Tumor*”, “Tumour*”, “Malignan*”, and “Carcinoma*”), as well as words of (“Lung*” OR “Pulmonary”) (Table S1). Moreover, the first 300 results of Google Scholar were checked manually as a gray literature search [48]. Backward and forward citation searches of all included studies were also performed.

### Study selection

All identified studies were exported to EndNote as the reference management software, and at first, every duplicated study was removed. Next, two researchers (LK and PS) independently screened the titles and abstracts of all the articles identified, using the inclusion criteria. The full texts of all studies that passed the first step were also reviewed by the same two researchers, and any disagreements were resolved via discussion or consultation with a third reviewer (SAN).

All types of clinical trials regardless of the classifications and stage of lung cancer with no age restrictions were included. Studies that were not clinical trials and did not investigate the effects of binimetinib on patients with lung cancer were excluded. Other types of studies, such as observational studies, review articles, and reanalysis of previously published studies, were excluded.

### Data extraction

Two reviewers (AJ and NAJ) independently extracted study characteristics from the original articles, including study title, first author’s name, country, publication date, trial phase, sampling setting, sample size, blinding status, study design, characteristics of participants (e.g. study population, sex, age, and race/ethnicity), binimetinib dose and schedule, duration of treatment and follow-up, other types of medications used, and main results, which were safety or efficacy outcomes. Any disagreements were resolved by discussion, and all the extracted data were double-checked by two other researchers (SAN and PS).

### Quality assessment

The risk of bias and quality of each of the selected studies were independently assessed by two independent reviewers (AJ and NAJ) using the National Institutes of Health (NIH) quality assessment tool for case series studies. There are nine domains: Domain 1: “Risk of bias arising from clear stating of study question or objectives”; Domain 2: “Risk of bias due to clear and complete describing of the study population”; Domain 3: “Risk of bias due to consecutiveness of cases”; Domain 4: “Risk of bias about the comparability of subjects”; Domain 5: “Risk of bias in clear describing of intervention”; Domain 6: “Risk of bias in the measurement of outcomes”; Domain 7: “Risk of bias due to satisfaction of length of follow-up”; Domain 8: “Risk of bias arising from illustrating obvious statistical methods”; and Domain 9: “Risk of bias due to complete reporting of results”, which finally concluded the overall risk of bias assessment in each study [49]. Disagreements were resolved by discussion between two reviewers or consulting with the principal investigator.

## Results

### Study selection

**Fig. 1:**
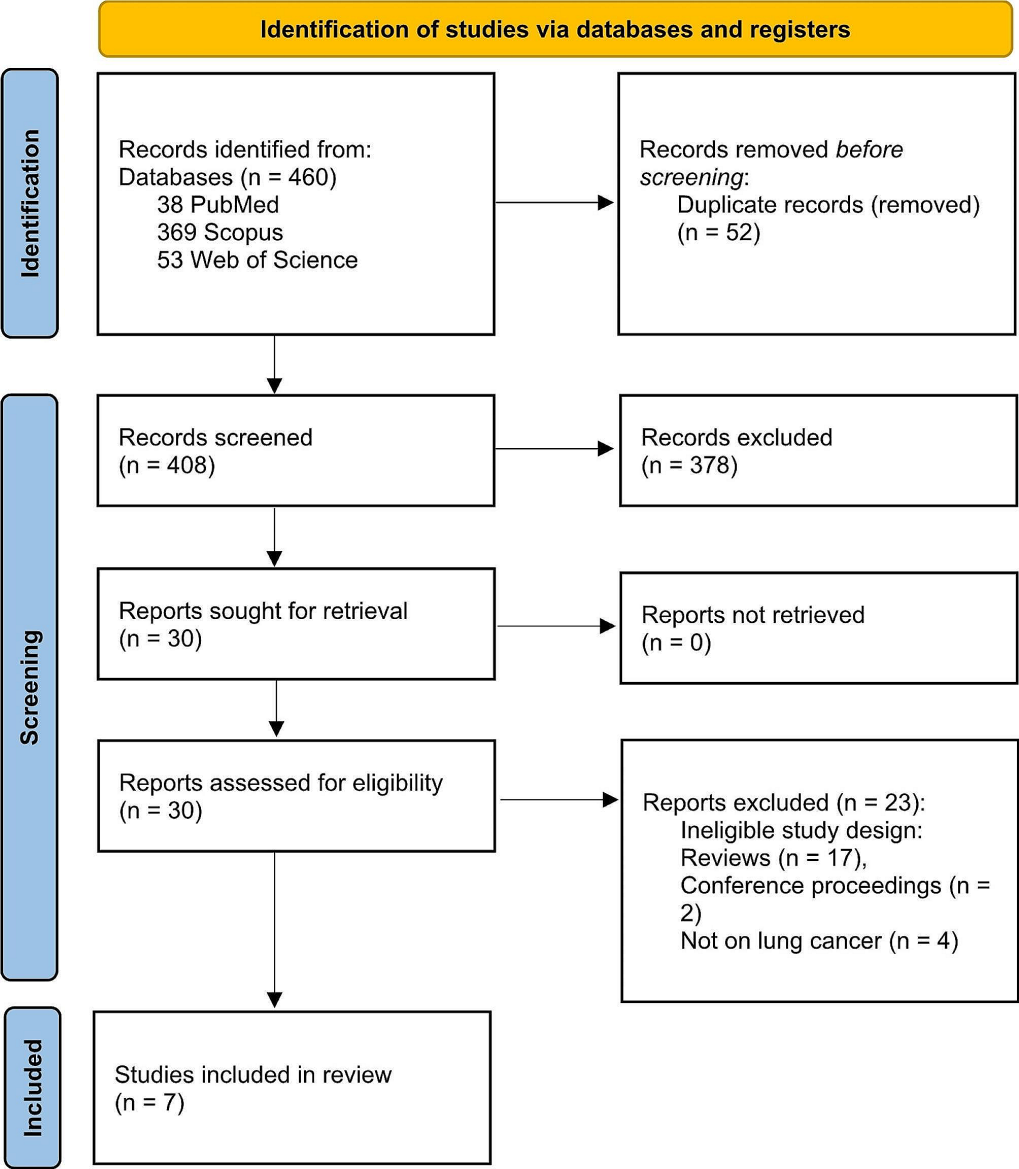
Study selection process

After excluding 52 duplicate search results from a total of 460 articles, 408 studies were included in the screening. Following the exclusion of 378 articles, 30 studies were eligible for full-text assessment. Among them, 23 reports were excluded because of inappropriate study designs, such as reviews (*n* = 17) [10, 50–65] or conference proceedings (*n* = 2) [66, 67], or because they were not conducted on patients with lung cancer (*n* = 4) [25, 68–70]. Finally, the remaining seven studies met the eligibility criteria and were included in our review [2, 3, 5, 33, 34, 71, 72] (Fig. 1).

### Quality assessment

All of the included studies had a low risk of bias because of the lack of clarity of the study question or objectives, the lack of clarity and completeness of the study population and the comparability of the subjects. In addition, the majority of the included studies (all but one [3]), had a low risk of bias in the measurement of outcomes and reporting of results. However, there were several concerns in several studies due to the consecutiveness of the patients, clear description of the intervention, and illustration of obvious statistical methods [5, 34, 71, 72]. Overall, four trials had good quality [2, 5, 33, 72], and three had fair quality judgment [3, 34, 71] (Table S2).

### Study characteristics

Among the included studies, there were three phase 2 [2, 33, 72] and four phase 1 [3, 5, 34, 71] studies, three of which were phase 1b [3, 34, 71]. All trials were open-label, single-arm publications published between 2020 and 2023. Two studies were multicenter and multinational [71, 72], one was conducted in 56 centers in five countries [72], two were from the United States [3, 33], one each from Canada [5], China [2], and Switzerland [34] (Table 1).

**Table 1:** Baseline characteristics of the included studies

Study ID	Study design and blinding	Country	Phase	Sample size			Study population	Total number of males (%)	Age (mean [SD] or median [range])	Length of treatment	Follow-up duration
				Total	Intervention	Control					
Riely et al. 2023 [72]	Single-arm open-label	Multicenter (56 centers in five countries)	II	98	59	39	Adult patients with histologically confirmed stage IV or recurrent NSCLC. Intervention: treatment-naive; Control: previously treated	46 (46.9%)	Intervention: 68 (47–83) Control: 71 (53–86)	Median 9.2 months (range, 0-35.1) Intervention: median 15.1 months (range, 0-35.1) Control: median 5.4 months (range, 0.1–31.2)	Intervention: Median 18.2 months (95% CI, 16.4–22.3) Control: 12.8 months (95% CI, 9.0-19.8)
Fung et al. 2021 [5]	Single-arm open-label	Canada	I	13	13	None	Adult patients with histologically confirmed stage IV non-squamous NSCLC	7 (53.8%)	57 (42–81)	Total median 5 cycles (range 1–18) for each patient	N/A
Saltos et al. 2023 [3]	Phase 1/1b open-label trial	United States	Ib	43	43	None	Stage IV NSCLC	23 (53%)	67 (32–80)	N/A	N/A
Zhou et al. 2022 [2]	An umbrella design open-label trial	China	II	22	22	None	Patients with stage IV adenocarcinoma	N/A	60.3 (SD = 6.1)	14.8 weeks	N/A
Bardia et al. 2020 [71]	Phase Ib open-label trial	Multi-country	Ib	27	27	None	Patients with advanced lung cancer	N/A	N/A	N/A	N/A
Froesch et al. 2021 [34]	Single-arm open-label	Switzerland	Ib	16	16	None	Patients with advanced, metastatic, or recurrent non-squamous NSCLC with KRAS exon 2 or 3 mutations	10 (63%)	60 (48–73)	N/A	N/A
Aggarwal et al. 2023 [33]	Single-arm phase II open-label trial	United States	II	9	9	None	Advanced KRAS-mutant NSCLC	5 (55.5%)	64 (52–77)	N/A	N/A

*Abbreviations* NSCLC: Non-small cell lung cancer; SD: Standard deviation; N/A: Not available; CI: Confidence interval

### Patient and lung cancer characteristics

In total, 228 patients were included in this review. One of the studies did not report the sex or age of the patients [71]; however 110 patients were male in six other studies. The median age of the participants was more than 60 years, with a median age of 71 years for the control group in the Riely et al. study [72]. The ascertainment of lung cancer was based on tumor samples [72], histology [2, 3, 5], or cytology [2] to identify cancer types. More than 95% of the included cases had adenocarcinoma, and approximately 2–5% of them had squamous cell carcinoma [2, 3, 33, 34, 72]. Five studies reported the cancer stage, and the metastatic form was the most common [2, 3, 5, 34, 72]. Among the seven studies, two reported advanced or recurrent KRAS-mutant NSCLC [33, 34], three reported stage IV NSCLC [3, 5, 72], one reported stage IV adenocarcinoma [2], and one reported non-specified advanced lung cancer [71]. Also, the median length of treatment was reported in three studies. In the Riely et al. study, the overall median duration of treatment with binimetinib was 8.4 months [72]. The median duration of treatment in the Zhou et al. study was 14.8 weeks [2]. According to Fung et al., there were 5 cycles (range 1–18) overall, 6.5 cycles (range 4–8) at dose level 1 (DL1), and 2 cycles (range 1–18) at DL2 [5] (Table 1). Table S3 shows the smoking patterns of the participants in each included study.

### Medication features

The oral dose of binimetinib was 30 [3] or 45 [2, 33, 71, 72] mg/BID. Some trials have used both dosages in different cycles [5, 34]; for example, Fung et al. used 30 mg/BID binimetinib for the first two weeks and 45 mg/BID for the next three weeks [5]. All included trials used adjuvant agents except for those of Zhou et al. [2]. The medicines used included oral encorafenib 450 mg once daily [72], carboplatin and pemetrexed 500 mg/m2 [5], oral erlotinib 100 mg daily [3], oral buparlisib 80 mg daily [71], cisplatin 75 mg/m2 and pemetrexed 500 mg/m2 [34], and oral hydroxychloroquine 400 mg/BID [33].

### Safety

All-cause adverse events (AEs) occurred in the majority of patients in all included studies, and more than 50% of these AEs were treatment-related AEs (TRAEs) [2, 3, 5, 33, 34, 71, 72]. The most commonly reported any grade AEs were diarrhea, fatigue, and nausea. Furthermore vomiting, dry skin or maculopapular rash, ocular toxicity, mucositis, edema, neutropenia, anorexia, symptomatic blood creatine phosphokinase (CPK) elevation, alanine aminotransferase (ALT) increase, anemia, hypertension, constipation, non-cardiac chest pain, and pruritus were other frequent TRAEs [2, 3, 5, 33, 34, 71, 72]. Generally, several serious AEs, such as colitis, diverticulitis, febrile neutropenia, pulmonary infection, anemia, dehydration, fever, hypoxia, pneumonia, lung infection, worsening of general condition, upper respiratory infection, arterial injury, and gastric ulcers, were reported in all the studies [2, 3, 5, 33, 34, 71, 72]. Moreover, five studies reported that some AEs led to dose limitations and drug discontinuation [2, 3, 5, 33, 71]. On the other hand, five studies reported different cases of death, which were mostly due to disease progression, pneumonia, cerebral metastases, and disseminated intravascular coagulation [2, 5, 33, 71, 72]. However, Riely et al. confirmed an intracranial hemorrhage-related death, which was assessed as a TRAE [72]. Also, nine (90%) and 17 (39.5%) cases in the Froesch et al. and Saltos et al. studies, respectively, presented at least one serious AE. Furthermore, 13 (100%) patients in the Fung et al. study, had at least one AE, including any grade (Table 2 and Table S4).

**Table 2:** Overall adverse events and treatment-related adverse events, by grade

Study ID	All-cause adverse events	TRAE of any grade	Grade 1 TRAE	Grade 2 TRAE	Grade 3 TRAE	Grade 4 TRAE	Grade 5 TRAE
Riely et al. 2023 [72]	97 (99%)	92 (94%)	N/A	N/A	37 (38%)	3 (3%)	N/A
Fung et al. 2021 [5]	N/A	N/A	N/A	N/A	DL1: anemia, neutropenia, and leukopenia (*n* = 1, 16.7% for each). Grade 4 neutropenia and thrombocytopenia (*n* = 1; 16.7%).		None
					DL2: grade 3: nausea, vomiting, diarrhea, ocular toxicity (reversible macular edema), fatigue, neutropenia, increased ALT, and increased serum amylase (*n* = 1, 14.3% for each). One patient had grade 3 seizures. No grade 4 toxicities documented at DL2.		
Saltos et al. 2023 [3]	97.7%	58%	N/A	N/A	N/A	None	
Zhou et al. 2022 [2]	Grade 3/4: 20 (91%)	37(56%)	N/A	N/A	12 (55%)		N/A
Bardia et al. 2020 [71]	N/A	Total: 83 (93.30%), grade 3/4:57 (64%)	N/A	N/A	Increased CPK (27%), increased ALT (14.6%), and increased AST (13.5%)		N/A
Froesch et al. 2021 [34]	N/A	9 out of 10 patients with chemotherapy and binimetinib	N/A	Fatigue (8; 50%), anorexia (5; 31%), nausea (5; 31%), anemia (4; 25%), hypertension (4; 25%), constipation (4; 25%), non-cardiac chest pain (3; 19%), pneumonitis (1; 6.25%)	Lung infection (4; 25%), fatigue (3; 19%), anemia (3; 19%), thromboembolic event (2; 12%), worsening of general condition (2; 12%), upper respiratory infection (1; 6%), arterial injury (1; 6%), gastric ulcer (1; 6%), and bone pain (1; 6%)	Decreased neutrophil count	N/A
Aggarwal et al. 2023 [33]	N/A	N/A	9 (100%)		5 (56%)		

*Abbreviations* TRAE: Treatment-related adverse event; N/A: Not available; DL: Dose level; ALT: Alanine aminotransferase; AST: Aspartate aminotransferase; CPK: Creatine phosphokinase

### Efficacy

Two studies reported the objective response rate (ORR) [5, 72] and median progression-free survival (PFS), and two others mentioned the number and type of mutations in participants (EGFRm and KRASm) [3, 71]. The ORR determined by independent radiology review (IRR) in Riely et al. was 75% for treatment-naive and 46% for previously treated patients [72]. Also, the ORR was 50%, according to an investigator-reported rate, and 33.3% disease control rate was reported in Fung et al. for twelve evaluable patients [5]. Furthermore, the median PFS was not estimable (NE) in the treatment-naive and 9.3 months in the previously treated participants in Riely et al., and the median PFS ranged from 1.9 to 9.6 months in the other studies [2, 3, 5, 33, 34, 71, 72]. Additionally, Riely et al. reported the median duration of response (DOR), NE in treatment-naive, and 16.7 months in previously treated arms, and Zhou et al. showed 5.5 months of median DOR [2, 72]. On the other hand, the disease control rate (DCR) after 24 weeks was 64% in treatment-naive and 41% in previously treated cases according to Riely et al., and ranged between 11.1% and 88.9% in other studies [2, 3, 5, 33, 34, 71, 72]. The overall survival (OS) ranged from 3.0 to 18.8 months in these studies [2, 3, 33, 34, 71]. Notably, Fung et al. reported that in participants with BRAFV600E-mutant metastatic NSCLC, the combination of encorafenib and binimetinib had significant antitumor activity [5] (Table 3).

**Table 3:** Efficacy of binimetinib for lung cancer

Study ID	ORR	Disease control	PFS	OS	DoR
Riely et al. 2023 [72]	Intervention: ORR by IRR: 75% (95% CI, 62–85) Investigator-assessed ORR: 63% (95% CI, 49–75) Control: ORR by IRR: 46% (95% CI, 30–63) Investigator-assessed ORR: 41% (95% CI, 26–58)	N/A	Intervention: Median by IRR: NE (95% CI, 15.7-NE) Control: 9.3 months (95% CI, 6.2-NE)	NE	Intervention: Median by IRR: NE (95% CI, 23.1-NE) Control: 16.7 months (95% CI, 7.4-NE)
Fung et al. 2021 [5]	Investigator-assessed: 50.0% (95% CI: 21.1-78.9%) Independent review: 33.3%	Investigator-assessed: 10 (83.3%) Independent review: 10 (83.3%).	N/A	N/A	N/A
Saltos et al. 2023 [3]	N/A	Total EGFRm (*n* = 17): N/A TKI-naïve EGFRm (*n* = 9): 88.90% EGFR-TKI pretreated EGFRm (*n* = 7): 12.50% KRASm (*n* = 22): 45.50%	Total EGFRm (*n* = 17): 7.8 months (1.8, 17.0) TKI-naïve EGFRm (*n* = 9) 9.6 months EGFR-TKI pretreated EGFRm (*n* = 7): N/A KRASm (*n* = 22): 7.6 months (4.4, 12.0)	Total EGFRm (*n* = 17): 15.2 months (9.5, 22.1) TKI-naïve EGFRm (*n* = 9): 18.8 months EGFR-TKI pretreated EGFRm (*n* = 7): N/A KRASm (*n* = 22): 3.0 months (1.6, 7.6)	N/A
Zhou et al. 2022 [2]	9.1%, 95% CI: 1–29%	59.1%, 95% CI: 36–79	3.8 months (1.6, 5.4)	9.2 months (3.5, 12.0)	5.5 months (3.6, 7.4)
Bardia et al. 2020 [71]	KRASm: 7.7%, 95% CI: 0.2–36.0 EGFRm: 0, 95% CI: 0.0-28.5	KRASm: 38.5%, 95% CI = 13.9–68.4 EGFRm: 54.5%	N/A	N/A	N/A
Froesch et al. 2021 [34]	29%	9 (65%)	5.7 months (1.1, 14.0)	6.5 months (1.8, NR)	N/A
Aggarwal et al. 2023 [33]	None	1 (11.1%)	1.9 months	5.3 months	N/A

*Abbreviations* ORR: Objective Response Rate; CI: confidence interval; PFS: Progression-Free Survival; OS: Overall Survival; IRR: independent radiology review; NE: not estimated; DoR: Duration of Response; NR: Not reported; N/A: Not available; EGFR: epidermal growth factor receptor; TKI: tyrosine kinase inhibitor

Disease control is defined as objective response + stable disease

## Discussion

We found that binimetinib alone or in combination with chemotherapy or other standard therapies was associated with a variety of TRAEs that led to dose reduction and drug discontinuation in most studies. Regarding the efficacy of binimetinib in terms of ORR, PFS, DOR, and OS, in most of the studies, there were no remarkable improvements in terms of antitumor activities. However, it should be noted that our included studies did not have a control arm, which makes comparison more difficult.

The use of MEK inhibitors as monotherapies or in combination with other drugs targeting the MAPK pathway has become a promising strategy for NSCLC patients with KRAS or BRAF mutations [73]. To date, four MEK inhibitors, trametinib, binimetinib, selumetinib, and cobimetinib, have been approved by the United States Food and Drug Administration, but only trametinib in combination with dabrafenib has been approved for the treatment of NSCLC patients with the BRAF V600E mutation [73]. The strategy of single therapy with MEK inhibitors may activate another parallel signaling pathway, that causes resistance to MEK inhibitors, and previous studies have shown that monotherapy with MEK inhibitors has lower efficacy and greater toxicity for NSCLC patients than chemotherapy alone [61]. Thus, it is more logical to use a combination of binimetinib plus other regimens for lung cancer treatment. In this regard, all of our included studies except for one [2] used other standard treatments like chemotherapy in combination with binimetinib.

The standard treatment for early stage NSCLC is surgery, and if unresectable, radiotherapy is recommended. Most NSCLC patients are diagnosed at advanced stages or with metastatic disease. The treatment of choice for advanced-stage NSCLC patients depends on multiple factors, such as the patient comorbidities, performance status, histology, and molecular features of the tumor, and includes surgery, radiotherapy, and chemotherapy alone or in combination with targeted therapy [74]. Other types of treatments have also been developed for lung cancer. In this regard, veliparib, which is a selective poly-(ADP)-ribose polymerase inhibitor is also used for lung cancer treatment; however, compared with chemotherapy, veliparib does not significantly improve patient outcomes in terms of efficacy and is associated with significantly greater AEs compared with chemotherapy [75]. The results of another systematic review of four studies on tislelizumab (i.e., an anti-programmed death-1 monoclonal antibody) revealed that it is almost safe and effective whether it is used in combination with chemotherapy or alone [76]. However, it should be noted that one of the major limitations of the abovementioned systematic review is the small sample size and number of included studies [76]. However, findings about combining MEK inhibitors with chemotherapeutic efficacy are controversial and probably depend on the tumor type and subtype; therefore, additional investigations should be performed to determine suitable combinations of MEK inhibitors with other chemotherapeutic and immunotherapeutic regimens for each candidate [61]. Currently, TKIs are the most common treatment strategy for relapsed small-cell lung cancer, followed by topoisomerase I inhibitors, immune checkpoint inhibitors, and alkylating agents [77]. As most of our studies focused on patients with NSCLC, further investigations of other novel treatments and other types of lung cancer are needed.

Our included studies showed that most of the patients experienced at least one of the AEs and they were mostly TRAEs. Most of the studies have shown manageable safety of the use of binimetinib as a monotherapy or in combination with other drugs [2, 3, 5, 33, 34, 71]. The minor differences between the studies using several types of drugs are due to the different mechanisms of action of the drugs, as well as their route of administration and elimination. Caunt and colleagues showed that MEK inhibitors in combination with BRAF inhibitors are better tolerated than are the respective monotherapies; however, all MEK inhibitors in combination with standard-of-care cytotoxic chemotherapy increase toxicity, and there is a need for dose reduction or dosing schedule alteration [78]. Furthermore, some treatments, such as the combination of bevacizumab and erlotinib, are associated with severe (grade ≥ 3) AEs [79]. Alternatively, the use of alectinib and crizotinib other than ceritinib as anaplastic lymphoma kinase (ALK) inhibitors, increased the risk of serious AEs compared with chemotherapy [80]. On the other hand, the safety profile of adjuvant EGFR-TKI therapy and MET inhibitors is generally manageable and tolerable [81, 82]. Immune checkpoint inhibitors also have a favorable safety profile [83], and their combinations with antiangiogenic agents have a better safety profile compared with combinations with chemotherapy [84]. It should be noted that both methods showed acceptable toxicity profiles in untreated or previously treated patients with advanced NSCLC [84]. Overall, it seems that patients with NSCLC, who received binimetinib, most likely experienced some sort of AEs, but serious AEs or death are unlikely to occur due to binimetinib, although this should be further investigated in future randomized controlled trials.

The efficacy measures that were most frequently reported in the included studies were OS, PFS, and ORR. Our systematic review revealed that the most efficient combination was binimetinib plus encorafenib in patients with BRAFV600-mutant metastatic NSCLC [72]. Only two studies showed modest efficacy [3, 5], and other combinations mostly had no significant efficacy [33, 34, 71]. A systematic review of ramucirumab in combination with docetaxel in patients with NSCLC showed that the pooled median of OS was 11.2 months and that the PFS was 5.7 months [85]. The same study revealed that the ORR ranged from 20.9 to 60.0%, and the DCR ranged from 62.4 to 90.0% [85]. The results of another meta-analysis on immunotherapy with programmed cell death protein 1/programmed cell death-ligand protein 1 (PD-1/PD-L1) inhibitor showed that the pooled ORR, DCR, OS, and PFS were 22.4%, 76.8%, 14.1 months, and 5.2 months, respectively [86]. Also, there were significantly better conditions in patients with EGFR-positive advanced NSCLC who received osimertinib than in controls in terms of efficacy measures [87]. In other studies, ALK inhibitors improved PSF compared with chemotherapy, and alectinib and brigatinib improved PFS compared with crizotinib and ceritinib [80]. In addition, alectinib improved OS compared with chemotherapy and crizotinib [80]. Another study confirmed the efficacy of crizotinib in NSCLC patients with ROS1 or MET gene mutations [88]. However, unlike crizotinib, some ALK inhibitors (e.g., lorlatinib, alectinib, and brigatinib) penetrate the central nervous system and are more effective in clinical studies [89]. In addition, in a study by Wang et al., lorlatinib was the best treatment option for patients with untreated ALK-positive advanced NSCLC [89]. Also, immune checkpoint inhibitors are useful treatment strategies for NSCLC patients compared with conventional treatment regimens, especially for patients who have achieved long-term tumor remission for more than two years with initial treatment lines [83, 90]. Immune checkpoint inhibitors combined with targeted personalized therapy reduced mortality in participants and has been confirmed as a first-line treatment for NSCLC based on efficacy and safety profile [83]. The combination of immunotherapy with antiangiogenic agents, with or without chemotherapy, has been demonstrated to promote antitumor activity in untreated or previously treated advanced NSCLC patients, resulting in promising and durable clinical benefits [84]. Other combinations, such as bevacizumab and erlotinib, have significantly improved PFS and ORR in metastatic NSCLC with EGFR mutations [79]. For patients with NSCLC with HER2 alterations, HER2-targeted therapy is considered an acceptable treatment strategy [91]. From indirect comparisons with other targeted therapies, it can be concluded that treatment with trastuzumab, deruxtecan, poziotinib, and pyrotinib is superior to chemotherapy [91]. MET inhibitors, especially savolitinib and tepotinib, are also promising treatment options for NSCLC [81]. Furthermore, in patients with completely resected early-stage NSCLC harboring mutated EGFR, adjuvant EGFR-TKI therapy may significantly prolong disease free survival as an important treatment option [82]. However, no impact on OS was observed compared with placebo or adjuvant chemotherapy [82]. Our systematic review showed that the ORR, median PFS, and OS were up to 75%, up to 9.3 months, and between 3.0 and 18.8 months, respectively. The differences between the abovementioned studies could be due to the use of several types of drugs with different mechanisms of action, the variations in the type of lung cancer (small cell lung cancer or NSCLC), the number of included studies, and their inclusion criteria (e.g., including only randomized controlled trials or non-randomized single-arm studies).

The doses of binimetinib regimens in the clinical trials included in our study were 30 mg or 45 mg BID. Despite all studies reporting a variety of AEs, including nausea, diarrhea, fatigue, vomiting, anemia, blurred vision, constipation, elevated ALT and AST levels, itching, and dry skin, it is important to note that the severity of these AEs varied, with some studies observing grade four or five AEs. However, due to the variety of concomitant medications used in these trials, it is challenging to accurately attribute these complications solely to binimetinib. Future meta-analyses should investigate the correlations between each dose of binimetinib and specific safety and efficacy outcomes to provide a clearer understanding of its risk-benefit profile.

Despite significant progress in the treatment outcomes of new lung cancer therapies, the cost of these drugs remains a major limiting factor for their acceptance by health payers worldwide. When evaluating economic analyses of new lung cancer therapies, the most important factors influencing the results are the comparator product selected, the perspective adopted, the scope of clinical benefit, the weighting of outcome benefits, and the cost or discount incurred in procuring the therapeutic active ingredient. Other important factors include the country of origin, as resource availability and treatment patterns can vary significantly across jurisdictions [92]. The cost-effectiveness thresholds of the studies also varied by study sponsor. Industry-provided costs increased over time and were significantly more dispersed compared to costs offered by non-profit sponsors [93]. For NSCLC patients, immunotherapy could be a cost-effective strategy in several scenarios [94]. On the other hand, the use of PD-L1 expression as a biomarker improves the cost-effectiveness of immunotherapy [94]. For example, the combination of PD-L1 and its overexpression with immune checkpoint inhibitors could be a cost-effective strategy to treat NSCLC with nivolumab as first-line and second-line treatments, as well as with pembrolizumab as first-line [95]. Molecular or biomarker testing and biomarker-based decision-making should therefore be included in the cost assessment, as they are an essential part of the personalized treatment of NSCLC. These offer patients a greater chance of increasingly effective treatment, minimizing AEs, and leading to an improved quality of life while optimizing the management of medical resources [92, 95]. The conclusions of some studies changed as the willingness-to-pay threshold increased. Cost-effectiveness decreases when the willingness-to-pay threshold falls below $100,000 per quality-adjusted life year. For example, pembrolizumab could be a cost-effective first-line treatment in NSCLC with a willingness-to-pay threshold set at $100,000 per quality-adjusted life year [94, 95]. Next research should perform the economic analysis or evaluate the cost-effectiveness of binimetinib in patients with lung cancer as the information could be valuable for policymakers and healthcare providers.

It is the pioneer systematic review to evaluate the safety and efficacy of binimetinib for lung cancer treatment and can guide further robust research on the clinical use of binimetinib as a MEK inhibitor in patients with lung cancer. However, this study has numerous limitations. Despite a comprehensive search of several databases and gray literature search, there is still a probability of missing some eligible studies. Also, the number of included studies was rather small. Due to the heterogeneity among the studies, which included distinct combination therapies plus binimetinib, a meta-analysis, a subgroup analysis, and publication bias assessment could not be conducted. The variation in study populations (e.g., stage IV or recurrent NSCLC, stage IV non-squamous NSCLC with or without KRAS mutations, and stage IV adenocarcinoma), treatment combinations (e.g., encorafenib, carboplatin, erlotinib, buparlisib, cisplatin or pemetrexed and hydroxychloroquine), and outcome measures (e.g., ORR, PFS, OS, DCR, and DOR) across the studies may impact the interpretation of results, limiting the generalizability of findings to broader patient populations. It is recommended to conduct future large-scale clinical trials. Then, updated systematic reviews can be performed with meta-analysis and subgroup analysis based on specific genetic mutations to provide insights into which patient populations might benefit most from binimetinib treatment. Additionally, the included studies were single-arm studies, and due to the lack of a control group, the results were not compared with any baseline data. Moreover, we focused only on binimetinib in this study and future systematic reviews could also consider other MEK inhibitors. The studies included according to the inclusion criteria mainly investigated the laboratory and histological aspects of patients and did not mention their quality of life, which can be considered in future research. We attempted to minimize selection and reviewer bias by using a comprehensive search strategy in several commonly used databases, without language or date restrictions, and a systematic approach to data extraction by including multiple reviewers to increase objectivity. We also addressed publication bias by including gray literature. Meanwhile, we addressed reporting bias by ensuring comprehensive data extraction. Furthermore, we assessed performance and detection bias by critically appraising study methods or assessing the risk of bias within individual studies and focusing on studies that used standardized assessment tools and reported outcome measures.

## Conclusions

Although binimetinib has shown some improvement in OS, PFS, and ORR, generally, its combination with chemotherapeutic agents has no significant effects. Several mild AEs can occur, but death is rare. According to the roles of different tumor types and adjuvant agents in medication efficacy, it is crucial to investigate the efficacy of different combinations of these agents with binimetinib for different tumor subtypes to identify more efficient and safer treatments for lung cancer patients. Further large-scale trials, especially randomized controlled trials, with diverse study populations in terms of age and type of NSCLC, as well as a thorough review of all outcome measures are recommended, to more precisely compare binimetinib with other MEK inhibitors or targeted therapies and their cost-effectiveness, safety, and efficacy.

### Electronic supplementary material

Below is the link to the electronic supplementary material.

Supplementary Material 1

Supplementary Material 2

## Data Availability

The data that supports the findings of this study are available in the supplementary material of this article.
