# Supplementary material for: The safety and efficacy of binimetinib for lung cancer: a systematic review
Source: BMC Pulm Med. 2024 Aug 1;24:379. doi: 10.1186/s12890-024-03178-4 (PMC11295668; doi:10.1186/s12890-024-03178-4)
Supplement: Supplementary file 1 — Supplementary Material 1 [file 12890_2024_3178_MOESM1_ESM.docx]

**Table S1.** Search strategy designed for four database

| **Database**  **(Search date)** | **Step** | **Search strategy** | **Number of results** |
| --- | --- | --- | --- |
| PubMed  (9.15.2023) | #1 | "binimetinib"[Supplementary Concept] OR “binimetinib”[tiab] OR “Mektovi”[tiab] OR “MEK162”[tiab] OR “MEK-162”[tiab] OR “MEK 162”[tiab] OR "ARRY 162"[tiab] OR “ARRY162"[tiab] OR "ARRY-162"[tiab] OR “ARRY-438162”[tiab] OR “ARRY 438162”[tiab] OR “ARRY438162”[tiab] OR “181R97MR71”[tiab] OR “MFCD22124525”[tiab] OR “CHEMBL3187723”[tiab] OR “NSC-764042”[tiab] OR “NSC-788187”[tiab] OR “NSC-799361”[tiab] OR “NCGC00345804-01”[tiab] OR “NCGC00345804-10”[tiab] | 384 |
|  | #2 | “Lung Neoplasms”[mh] OR “Pulmonary Blastoma”[mh] OR ((“Lung”[mh] OR “Lung*”[tiab] OR “Pulmonary”[tiab]) AND (“Neoplasms”[mh] OR “Neoplas*”[tiab] OR “Cancer*”[tiab] OR “Tumor*”[tiab] OR “Tumour*”[tiab] OR “Malignan*”[tiab] OR “Carcinoma*”[tiab] OR “Carcinoid*” OR “Adenocarcinoma*”[tiab] OR “Lymphoma*”[tiab] OR “Sarcoma*”[tiab] OR “blastoma*”[tiab] OR “Fibrosarcoma*”[tiab] OR “Leiomyosarcoma*”[tiab])) | 497,962 |
|  | #3 | #1 AND #2 | 38 |
| Scopus  (9.15.2023) | #1 | TITLE-ABS-KEY (“binimetinib” OR “Mektovi” OR “MEK162” OR “MEK-162” OR “MEK 162” OR "ARRY 162" OR “ARRY162" OR "ARRY-162" OR “ARRY-438162” OR “ARRY 438162” OR “ARRY438162” OR “181R97MR71” OR “MFCD22124525” OR “CHEMBL3187723” OR “NSC-764042” OR “NSC-788187” OR “NSC-799361” OR “NCGC00345804-01” OR “NCGC00345804-10”) | 1696 |
|  | #2 | TITLE-ABS-KEY ((“Lung*” OR “Pulmonary”) AND (“Neoplas*” OR “Cancer*” OR “Tumor*” OR “Tumour*” OR “Malignan*” OR “Carcinoma*” OR “Carcinoid*” OR “Adenocarcinoma*” OR “Lymphoma*” OR “Sarcoma*” OR “blastoma*” OR “Fibrosarcoma*” OR “Leiomyosarcoma*”) ) | 738,377 |
|  | #3 | #1 AND #2 | 369 |
| Web of Science  (9.15.2023) | #1 | TS=(“binimetinib” OR “Mektovi” OR “MEK162” OR “MEK-162” OR “MEK 162” OR "ARRY 162" OR “ARRY162" OR "ARRY-162" OR “ARRY-438162” OR “ARRY 438162” OR “ARRY438162” OR “181R97MR71” OR “MFCD22124525” OR “CHEMBL3187723” OR “NSC-764042” OR “NSC-788187” OR “NSC-799361” OR “NCGC00345804-01” OR “NCGC00345804-10”) | 521 |
|  | #2 | TS=((“Lung*” OR “Pulmonary”) AND (“Neoplas*” OR “Cancer*” OR “Tumor*” OR “Tumour*” OR “Malignan*” OR “Carcinoma*” OR “Carcinoid*” OR “Adenocarcinoma*” OR “Lymphoma*” OR “Sarcoma*” OR “blastoma*” OR “Fibrosarcoma*” OR “Leiomyosarcoma*”)) | 516,108 |
|  | #3 | #1 AND #2 | 53 |
| Google Scholar  (9.24.2023) |  | ("binimetinib" OR “Mektovi”) AND (“Lung Cancer” OR “Pulmonary Cancer”) | About 4,500 |

**Table S2.** Revised National Institutes of Health (NIH) quality assessment tool results for studies.

| Study ID | 1 | 2 | 3 | 4 | 5 | 6 | 7 | 8 | 9 | Overall quality |
| --- | --- | --- | --- | --- | --- | --- | --- | --- | --- | --- |
| Riely et al. 2023 (1) | Y | Y | N | Y | Y | Y | N | Y | Y | 7 (Good) |
| Fung et al. 2021 (2) | Y | Y | N | Y | Y | Y | Y | Y | Y | 8 (Good) |
| Saltos et al. 2023 (3) | Y | Y | Y | Y | Y | N | CD | Y | N | 6 (Fair) |
| Zhou et al. 2022 (4) | Y | Y | Y | Y | Y | Y | CD | Y | Y | 8 (Good) |
| Bardia et al. 2020 (5) | Y | Y | Y | Y | N | Y | CD | N | Y | 6 (Fair) |
| Froesch et al. 2021 (6) | Y | Y | Y | Y | N | Y | CD | N | Y | 6 (Fair) |
| Aggarwal et al. 2023 (7) | Y | Y | Y | Y | Y | Y | CD | Y | Y | 8 (Good) |

Abbreviations: Y: Yes, N: No; and CD: cannot determine.

Domain 1**:** Was the study question or objective clearly stated?

Domain 2: Was the study population clearly and fully described, including a case definition?

Domain 3: Were the cases consecutive?

Domain 4: Were the subjects comparable?

Domain 5: Was the intervention clearly described?

Domain 6: Were the outcome measures clearly defined, valid, reliable, and implemented consistently across all study participants?

Domain 7: Was the length of follow-up adequate?

Domain 8: Were the statistical methods well-described?

Domain 9: Were the results well-described?

Overall quality rating: Good: 7-9; Fair: 4-6; and Poor: 0-3

**Table S3.** Characteristics of smoking patterns among the included participant in each included study.

| **Study ID** | **Sample size** | **Smoking Status** | | | | | | | |
| --- | --- | --- | --- | --- | --- | --- | --- | --- | --- |
|  |  | **Intervention (N, Percent)** | | | | **Control (N, Percent)** | | | |
|  |  | **Former** | **Current** | **Never** | **Missing** | **Former** | **Current** | **Never** | **Missing** |
| Riely et al. 2023 (1) | 98 | 33, 56% | 8, 14% | 18, 31% | 0, 0% | 23, 59% | 5, 13% | 11, 28% | 0, 0% |
| Fung et al. 2021 (2) | 13 | 3, 23% | 3, 23% | 7, 53.8% | 0, 0% | No Control | No Control | No Control | No Control |
| Saltos et al. 2023 (3) | 43 | 27, 63% | 6, 14% | 10, 23% | N/A | No Control | No Control | No Control | No Control |
| Zhou et al. 2022 (4) | 22 | N/A | N/A | N/A | N/A | No Control | No Control | No Control | No Control |
| Bardia et al. 2020 (5) | 27 | N/A | N/A | N/A | N/A | No Control | No Control | No Control | No Control |
| Froesch et al. 2021 (6) | 16 | 9, 56% | 4, 25% | 1, 6% | 2, 13% | No Control | No Control | No Control | No Control |
| Aggarwal et al. 2023 (7) | 9 | N/A | 7, 78% | N/A | N/A | No Control | No Control | No Control | No Control |

**Table S4.** Reported adverse events of the included studies.

| **Study ID** | Riely et al. 2023 (1) | Fung et al. 2021 (2) | Saltos et al. 2023 (3) | Zhou et al. 2022 (4) | Bardia et al. 2020 (5) | Froesch et al. 2021 (6) | Aggarwal et al. 2023 (7) |
| --- | --- | --- | --- | --- | --- | --- | --- |
| **Serious adverse events** | N/A | N/A | 17 (39.5%); most common ones:  diarrhea (n=4), dehydration (n=2), fever (n=2), and hypoxia (n=2) | 32 (49%) | N/A | N/A | 4(44%) |
|  |  |  |  |  |  |  |  |
|  |  |  |  |  |  |  |  |
| **Death** | 30 (31%) due to disease progression (24%), AE (2%), or other causes (4%). | N=1 | N/A | No deaths were suspected to be study drug related. | N/A | N/A | None |
|  |  |  |  |  |  |  |  |
| **Nausea** | Any grade: 49 (50%)  Grade 3: 3 (3%)  Grade 4: 0 | Any grade: 6 (46.1%)  Grade 3/4: 1 (7.7%) | Grade 1: 28; grade 2: 5, and grade 3: 2 | 3 (14%) | Total: 32(36%); grade 2: 5 (31%), and grade 3: 2 (12%) | 3(14%) | N/A |
| **Diarrhea** | Aby grade: 42 (43%); grade 3: 4 (4%) and grade 4: 0 | Any grade: 6 (46.1%) and grade 3/4: 1 (7.7%) | Grade 2 and 3: 12 | 11 (50%) | Total: 51(57.3%), grade 3/4=7(7.9%) | Grade 2: 2 (12%); grade 3: 1 (6%), grade 4: 0 | Grade 1-2: 4 (44%) and grade 3: 1 (11%) |
| **Blurred vision** | Any Grade  17 (17)  Grade 3  1 (1)  Grade 4  0 | N/A | N/A | N/A | N/A | N/A | N/A |
| **Constipation** | Any Grade  13 (13)  Grade 3  0  Grade 4  0 | N/A | N/A | N/A | N/A | Grade 2  4(25%)  Grade 3  0(0%)  Grade 4  0(0%) | N/A |
| **Increased ALT** | Any Grade  12 (12)  Grade 3  5 (5)  Grade 4  0 | Any grade  1 (7.7)  Grade 3/4  1 (7.7) | Grade 1: 7, grade 2 and 3: 0 | 3(14%) | All=32(36%), grade 3/4=13(14.6%) | N/A | Grade 1-2  1(11%)  Grade 3  0(0%) |
| **Increased AST** | Any Grade  12 (12)  Grade 3  7 (7)  Grade 4  0 | Any grade  1 (7.7)  Grade 3/4  0 | N/A | N/A | All=44(49.4%), grade 3/4=12(13.5%) | N/A | Grade 1-2  0(0%)  Grade 3  1(11%) |
| **Fatigue** | Any Grade  31 (32)  Grade 3  2 (2)  Grade 4  0 | 6 (46.)  1 (7.7) | Grade 1: 16; grade 2: 14; and grade 3: 2 | N/A | All=23(25.8%), grade 3/4=1(1.1%) | Grade 2  8(50%)  Grade 3  3(19%)  Grade 4  0(0%) | Grade 1-2  3(33%)  Grade 3  0(0%) |
| **Vomiting** | Any Grade  28 (29)  Grade 3  1 (1)  Grade 4  0 | Any grade  2 (15.4)  Grade 3/4  1 (7.7) | Grade 1: 12; grade 2: 0; and grade 3: 2 | 5(23%) | All=22(24.7%), grade 3/4=0(0%) | Grade 2  1(6%)  Grade 3  1(6%)  Grade 4  0(0%) | N/A |
| **Anemia** | Any Grade  18 (18)  Grade 3  3 (3)  Grade 4  0 | Any grade  2 (15.4)  Grade 3/4  1 (7.7) | Grade 1: 0; grade 2: 0; and grade 3: 2 | 3(14%) | N/A | Grade 2  4(25%)  Grade 3  3(19%)  Grade 4  0(0%) | N/A |
| **Asthenia** | Any Grade  10 (10)  Grade 3  3 (3)  Grade 4  0 | N/A | N/A | 2(9%) | N/A | N/A | N/A |
| **Dry skin** | Any Grade  10 (10)  Grade 3  3 (3)  Grade 4  0 | N/A | Grade 1: 9; grade 2: 0; and grade 3: 0 | N/A | All=9(10.1%), grade 3/4=1(1.1%) | N/A | N/A |
| **Dry skin/Rash** | N/A | Any grade  10 (76.9)  Grade 3/4  0 | N/A | 15(68%) | All=28(31.5%), grade 3/4=4(4.5%) | N/A | N/A |
| **Rash/Acne** | N/A | N/A | Grade 1: 35; grade 2: 23; and grade 3: 9 | N/A | N/A | N/A | Grade 1-2  3(33%)  Grade 3  3(33%) |
| **Peripheral edema** | Any Grade  11 (11)  Grade 3  0  Grade 4  0 | N/A | N/A | 2(9%) | All=14(15.7%), grade 3/4=0(0%) | Grade 2  2(12%)  Grade 3  0(0%)  Grade 4  0(0%) | N/A |
| **Abdominal pain** | Any Grade  10 (10)  Grade 3  0  Grade 4  0 | N/A | N/A | 2(9%)  Upper abdominal pain: 2(9%) | N/A | N/A | N/A |
| **Alopecia** | Any Grade  10 (10)  Grade 3  0  Grade 4  0 | N/A | N/A | N/A | N/A | Grade 2  1(6%)  Grade 3  0(0%)  Grade 4  0(0%) | N/A |
| **Pruritus** | Any Grade  12 (12)  Grade 3  0  Grade 4  0 | Any grade  1 (7.7)  Grade 3/4  0 | N/A | 3(14%) | All=12(13.5%), grade 3/4=1(1.1%) | N/A | Grade 1-2  3(33%)  Grade 3  0(0%) |
| **Increased CRP** | Any Grade  11 (11)  Grade 3  0  Grade 4  0 | Any grade  1 (7.7)  Grade 3/4  0 | Grade 1: 16; grade 2: 7; and grade 3: 0 | 16(73%) | All=53(59.6%), grade 3/4=24(27%) | Grade 2  1(6%)  Grade 3  1(6%)  Grade 4  0(0%) | N/A |
| **Increased bilirubin** | N/A | Any grade  1 (7.7)  Grade 3/4  0 | Grade 1: 5; grade 2: 2; and grade 3: 0 | N/A | N/A | N/A | N/A |
| **Dysuria/ Hematuria** | N/A | Any grade  1 (7.7)  Grade 3/4  0 | N/A | N/A | N/A | N/A | N/A |
| **Edema** | N/A | Any grade  5 (38.5)  Grade 3/4  0 | N/A | N/A | N/A | N/A | N/A |
| **Anorexia** | N/A | Any grade  1 (7.7)  Grade 3/4  0 | Grade 1: 21; grade 2: 2; and grade 3: 0 | N/A | N/A | Grade 2  5(31%)  Grade 3  0(0%)  Grade 4  0(0%) | N/A |
| **Bilateral leg weakness** | N/A | Any grade  1 (7.7)  Grade 3/4  0 | N/A | N/A | N/A | N/A | N/A |
| **Hypokalemia** | N/A | Any grade  1 (7.7)  Grade 3/4  0 | N/A | N/A | N/A | N/A | N/A |
| **Joint pain** | N/A | Any grade 2 (15.4); Grade 3/4 0 | N/A | N/A | N/A | N/A | N/A |
| **Mucositis** | N/A | Any grade  8 (61.5)  Grade 3/4  0 | Grade 1: 9; grade 2: 2; and grade 3: 0 | N/A | N/A | Grade 2  0(0%)  Grade 3  0(0%)  Grade 4  1(6%) | N/A |
| **Decreased neutrophil count** | N/A | Any grade  4 (30.8)  Grade 3/4  2 (15.4) | N/A | None | N/A | N/A | N/A |
| **Ocular toxicity** | N/A | Any grade  8 (61.5)  Grade 3/4  1 (7.7) | N/A | N/A | N/A | N/A | N/A |
| **Palpitation/ Sinus tachycardia** | N/A | Any grade: 2 (15.4); Grade 3/4: 0 | N/A | N/A | N/A | N/A | N/A |
| **Paronychia** | N/A | Any grade  1 (7.7)  Grade 3/4  0 | Grade 1: 5; grade 2: 2; and grade 3: 0 | N/A | N/A | N/A | N/A |
| **Weight gain** | N/A | Any grade  1 (7.7)  Grade 3/4  0 | N/A | N/A | N/A | N/A | N/A |
| **Weight loss** | N/A | Any grade  1 (7.7)  Grade 3/4  0 | N/A | N/A | N/A | Grade 2: 1(6%) Grade 3: 0(0%); Grade 4: 0(0%) | N/A |
| **Decreased leukocyte** | N/A | Any grade  1 (7.7)  Grade 3/4  1 (7.7) | N/A | N/A | N/A | N/A | N/A |
| **Increased amylase** | N/A | Any grade  1 (7.7)  Grade 3/4  1 (7.7) | N/A | 5(23%) | All=17(19.1%), grade 3/4=4(4.5%) | N/A | N/A |
| **Vertigo** | N/A | Any grade  1 (7.7)  Grade 3/4  0 | N/A | N/A | N/A | N/A | N/A |
| **Decreased ejection fraction** | N/A | Any grade  1 (7.7)  Grade 3/4  0 | Grade 1: 2; grade 2: 0; and grade 3: 9 | N/A | N/A | N/A | N/A |
| **Epistaxis** | N/A | Any grade  1 (7.7)  Grade 3/4  0 | N/A | N/A | N/A | N/A | N/A |
| **Decreased platelet count** | N/A | N/A | N/A | N/A | N/A | Grade 2  1(6%)  Grade 3  0(0%)  Grade 4  0(0%) | N/A |
| **Generalized weakness** | N/A | N/A | Grade 1: 5; grade 2: 9; and grade 3: 0 | N/A | N/A | Grade 2  1(6%)  Grade 3  1(6%)  Grade 4  0(0%) | N/A |
| **Eye disorder** | N/A | N/A | Grade 1: 5; grade 2: 5; and grade 3: 2 | N/A | N/A | N/A | N/A |
| **Dehydration** | N/A | N/A | Grade 1: 0; grade 2: 5; and grade 3: 5 | N/A | N/A | Grade 2  1(6%)  Grade 3  0(0%)  Grade 4  0(0%) | N/A |
| **QT prolongation** | N/A | N/A | Grade 1: 5; grade 2: 2; and grade 3: 2 | 5(23%) | N/A | N/A | N/A |
| **Hypomagnesemia** | N/A | N/A | Grade 1: 9; grade 2: 0; and grade 3: 0 | N/A | N/A | N/A | N/A |
| **Dry mouth** | N/A | N/A | Grade 1: 5; grade 2: 0; and grade 3: 0 | N/A | N/A | N/A | N/A |
| **Mouth ulcer** | N/A | N/A | N/A | 8(36%) | N/A | N/A | N/A |
| **Increased troponin** | N/A | N/A | Grade 1: 2; grade 2: 0; and grade 3: 2 | N/A | N/A | N/A | N/A |
| **Myalgia** | N/A | N/A | Grade 1: 2; grade 2: 2; and grade 3: 0 | N/A | N/A | N/A | N/A |
| **Hyperglycemia** | N/A | N/A | Grade 1: 0; grade 2: 0; and grade 3: 2 | N/A | All=17(19.1%), grade 3/4=0(0%) | N/A | N/A |
| **Syncope** | N/A | N/A | Grade 1: 0; grade 2: 0; and grade 3: 2 | N/A | N/A | N/A | N/A |
| **Pleural effusion** | N/A | N/A | Grade 1: 0; grade 2: 0; and grade 3: 2 | N/A | N/A | N/A | N/A |
| **Increased GGT** | N/A | N/A | N/A | 2(9%) | N/A | Grade 2: 1(6%); Grade 3: 0(0%); Grade 4: 0(0%) | N/A |
| **Increased ALP** | N/A | N/A | N/A | 3(14%) | N/A | N/A | N/A |
| **Decreased appetite** | N/A | N/A | N/A | 4(18%) | All=20(22.5%), grade 3/4=0(0%) | N/A | N/A |
| **Stomatitis** | N/A | N/A | N/A | N/A | All=33(37.1%), grade 3/4=6(6.7%) | N/A | N/A |
| **Maculopapular rash** | N/A | N/A | N/A | N/A | All=30(33.7%), grade 3/4=11(12.4%) | Grade 2  1(6%)  Grade 3  1(6%)  Grade 4  0(0%) | N/A |
| **Dermatitis acneiform** | N/A | N/A | N/A | N/A | All=26(29.2%), grade 3/4=4(4.5%) | N/A | N/A |
| **Chorioretinopathy** | N/A | N/A | N/A | N/A | All=16(18%), grade 3/4=0(0%) | N/A | N/A |
| **Increased lipase** | N/A | N/A | N/A | 2(9%) | All=16(18%), grade 3/4=7(7.9%) | N/A | N/A |
| **Leukocytosis** | N/A | N/A | N/A | 3(14%) | N/A | Grade 2  0(0%)  Grade 3  1(6%)  Grade 4  0(0%) | N/A |
| **Acute kidney injury** | N/A | N/A | N/A | 4(18%) | N/A | Grade 2  1(6%)  Grade 3  0(0%)  Grade 4  0(0%) | N/A |
| **Arterial injury** | N/A | N/A | N/A | N/A | N/A | Grade 2  0(0%)  Grade 3  1(6%)  Grade 4  0(0%) | N/A |
| **Back pain** | N/A | N/A | N/A | N/A | N/A | Grade 2  1(6%)  Grade 3  0(0%)  Grade 4  0(0%) | N/A |
| **Bone pain** | N/A | N/A | N/A | N/A | N/A | Grade 2  0(0%)  Grade 3  1(6%)  Grade 4  0(0%) | N/A |
| **Conjunctivitis** | N/A | N/A | N/A | N/A | N/A | Grade 2  1(6%)  Grade 3  0(0%)  Grade 4  0(0%) | N/A |
| **Cough** | N/A | N/A | N/A | 2(9%) | N/A | Grade 2  1(6%)  Grade 3  0(0%)  Grade 4  0(0%) | N/A |
| **Increased creatinine** | N/A | N/A | N/A | N/A | N/A | Grade 2  2(12%)  Grade 3  0(0%)  Grade 4  0(0%) | N/A |
| **Depression** | N/A | N/A | N/A | N/A | N/A | Grade 2  1(6%)  Grade 3  0(0%)  Grade 4  0(0%) | N/A |
| **Dizziness** | N/A | N/A | N/A | N/A | N/A | Grade 2  2(12%)  Grade 3  0(0%)  Grade 4  0(0%) | N/A |
| **Duodenal ulcer** | N/A | N/A | N/A | N/A | N/A | Grade 2  0(0%)  Grade 3  1(6%)  Grade 4  0(0%) | N/A |
| **Face edema** | N/A | N/A | N/A | N/A | N/A | Grade 2  1(6%)  Grade 3  0(0%)  Grade 4  0(0%) | N/A |
| **Dyspnea** | N/A | N/A | N/A | N/A | N/A | Grade 2  0(0%)  Grade 3  1(6%)  Grade 4  0(0%) | N/A |
| **Esophageal fistula** | N/A | N/A | N/A | N/A | N/A | Grade 2  0(0%)  Grade 3  1(6%)  Grade 4  0(0%) | N/A |
| **Esophageal stent insertion** | N/A | N/A | N/A | N/A | N/A | Grade 2  0(0%)  Grade 3  1(6%)  Grade 4  0(0%) | N/A |
| **Flu like symptoms** | N/A | N/A | N/A | N/A | N/A | Grade 2  1(6%)  Grade 3  0(0%)  Grade 4  0(0%) | N/A |
| **Gastric ulcer** | N/A | N/A | N/A | N/A | N/A | Grade 2  0(0%)  Grade 3  1(6%)  Grade 4  0(0%) | N/A |
| **Headache** | N/A | N/A | N/A | N/A | N/A | Grade 2  2(12%)  Grade 3  0(0%)  Grade 4  0(0%) | N/A |
| **Hypercalcemia** | N/A | N/A | N/A | N/A | N/A | Grade 2  1(6%)  Grade 3  0(0%)  Grade 4  0(0%) | N/A |
| **Hypertension** | N/A | N/A | N/A | N/A | N/A | Grade 2  4(25%)  Grade 3  2(12%)  Grade 4  0(0%) | N/A |
| **Hypocalcemia** | N/A | N/A | N/A | N/A | N/A | Grade 2  1(6%)  Grade 3  1(6%)  Grade 4  0(0%) | N/A |
| **Hypalbuminemia** | N/A | N/A | N/A | N/A | N/A | Grade 2  1(6%)  Grade 3  0(0%)  Grade 4  0(0%) | N/A |
| **Hyponatremia** | N/A | N/A | N/A | N/A | N/A | Grade 2  0(0%)  Grade 3  2(12%)  Grade 4  0(0%) | N/A |
| **Hypoxia** | N/A | N/A | N/A | N/A | N/A | Grade 2  0(0%)  Grade 3  1(6%)  Grade 4  0(0%) | N/A |
| **Lung infection** | N/A | N/A | N/A | N/A | N/A | Grade 2  0(0%)  Grade 3  4(25%)  Grade 4  0(0%) | N/A |
| **Non-cardiac chest pain** | N/A | N/A | N/A | N/A | N/A | Grade 2  3(19%)  Grade 3  0(0%)  Grade 4  0(0%) | N/A |
| **Oral dysesthesia** | N/A | N/A | N/A | N/A | N/A | Grade 2  1(6%)  Grade 3  0(0%)  Grade 4  0(0%) | N/A |
| **Pneumonitis** | N/A | N/A | N/A | N/A | N/A | Grade 2  1(6%)  Grade 3  0(0%)  Grade 4  0(0%) | N/A |
| **Presyncope** | N/A | N/A | N/A | N/A | N/A | Grade 2  1(6%)  Grade 3  0(0%)  Grade 4  0(0%) | N/A |
| **Papulopustular rash** | N/A | N/A | N/A | N/A | N/A | Grade 2  2(12%)  Grade 3  0(0%)  Grade 4  0(0%) | N/A |
| **Retinopathy** | N/A | N/A | N/A | N/A | N/A | Grade 2  0(0%)  Grade 3  1(6%)  Grade 4  0(0%) | N/A |
| **Thoracic pain** | N/A | N/A | N/A | N/A | N/A | Grade 2  0(0%)  Grade 3  1(6%)  Grade 4  0(0%) | N/A |
| **Thromboembolic events** | N/A | N/A | N/A | N/A | N/A | Grade 2  0(0%)  Grade 3  2(12%)  Grade 4  0(0%) | N/A |
| **Upper respiratory infection** | N/A | N/A | N/A | N/A | N/A | Grade 2  1(6%)  Grade 3  1(6%)  Grade 4  0(0%) | N/A |
| **Urinary tract infection** | N/A | N/A | N/A | N/A | N/A | Grade 2  0(0%); Grade 3  1(6%); Grade 4  0(0%) | N/A |

Abbreviations: N/A: Not available; DL: Dose level; ALT: Alanine aminotransferase; AST: Aspartate aminotransferase; CPK: Creatine phosphokinase; GGT: gamma-glutamyl transferase; ALP: alkaline phosphatase.

**References**

|  |
| --- |

1. Riely GJ, Smit EF, Ahn M-J, Felip E, Ramalingam SS, Tsao A, et al. Phase II, open-label study of encorafenib plus binimetinib in patients with BRAF V600-mutant metastatic non–small-cell lung cancer. Journal of Clinical Oncology. 2023:JCO. 23.00774.

2. Fung AS, Graham DM, Chen EX, Stockley TL, Zhang T, Le LW, et al. A phase I study of binimetinib (MEK 162), a MEK inhibitor, plus carboplatin and pemetrexed chemotherapy in non-squamous non-small cell lung cancer. Lung Cancer. 2021;157:21-9.

3. Saltos AN, Creelan BC, Tanvetyanon T, Chiappori AA, Antonia SJ, Shafique MR, et al. A phase I/IB trial of binimetinib in combination with erlotinib in NSCLC harboring activating KRAS or EGFR mutations. Lung Cancer. 2023;183.

4. Zhou Q, Chen H-J, Wang B-C, Wang Z, Tu H-Y, Xu C-R, et al. CLUSTER: A biomarker-integrated targeted therapy study in patients with advanced non-small cell lung cancer. 2022.

5. Bardia A, Gounder M, Rodon J, Janku F, Lolkema MP, Stephenson JJ, et al. Phase Ib Study of Combination Therapy with MEK Inhibitor Binimetinib and Phosphatidylinositol 3-Kinase Inhibitor Buparlisib in Patients with Advanced Solid Tumors with RAS/RAF Alterations. Oncologist. 2020;25(1):e160-e9.

6. Froesch P, Mark M, Rothschild SI, Li Q, Godar G, Rusterholz C, et al. Binimetinib, pemetrexed and cisplatin, followed by maintenance of binimetinib and pemetrexed in patients with advanced non-small cell lung cancer (NSCLC) and KRAS mutations. The phase 1B SAKK 19/16 trial. Lung Cancer. 2021;156:91-9.

7. Aggarwal C, Maity AP, Bauml JM, Long Q, Aleman T, Ciunci C, et al. A Phase II Open-Label Trial of Binimetinib and Hydroxychloroquine in Patients With Advanced KRAS-Mutant Non-Small Cell Lung Cancer. Oncologist. 2023;28(7):644-E564.
